# The effect of a smartphone-based pain management application on pain intensity and quality of life in adolescents with chronic pain

**DOI:** 10.1038/s41598-021-86156-8

**Published:** 2021-03-23

**Authors:** Maryam Shaygan, Azita Jaberi

**Affiliations:** Community Based Psychiatric Care Research Center, School of Nursing and Midwifery, Shiraz University of Medical Sciences, Shiraz, Iran

**Keywords:** Psychology, Health care, Medical research

## Abstract

The development, implementation, and qualitative evaluation of smartphone-based pain management applications may provide an opportunity for more optimal management of pediatric pain in the homesetting. The present mixed-method study was conducted to assess a smartphone-based pain management application regarding the feasibility, adherence, participant satisfaction, and effectiveness on pain intensity and quality of life in adolescents with chronic pain. The study was carried out in the quantitative and qualitative stages using a mixed-method approach. The quantitative stage included 128 adolescents who met the ICD-11 criteria of chronic pain. After random allocation, adolescents allocated to the intervention group received a pain management program through a smartphone-based application. No education was given to the adolescents in the control group. The adolescents were assessed regarding pain intensity and different dimensions of quality of life at pre-intervention, post-intervention, and three-month follow-up. The findings in the quantitative stage were explained by qualitative interviews. The findings of the quantitative stage showed significant improvements in the pain intensity, emotional, social, and school functioning but not in the physical functioning of the adolescents. A high level of adherence (78.12%) and satisfaction (Mean = 26.45, SD = 6.45) with thes martphone-based pain management program was found. Based on the qualitative interviews, adolescents’ pain management strategies can be classified in three main categories: physical management, psychological management, and interpersonal resources. The results confirm the positive effect of a smartphone-based pain management program on the pain intensity and different dimensions of quality of life of adolescents with chronic pain. Within the context of chronic pain management, a mobile application incorporating both the psychological and physical management of pain may help adolescents with chronic pain to reduce the negative impacts of pain on their life.

## Introduction

Pediatric chronic pain (i.e., pain lastinglonger than 3 months) is a serious developmental health concern leading toconsiderable physical and psychological consequences, as well as having high clinical, social, and economic burden^1,2^. One recent study among adolescents across 42 countries demonstrated that on average 20.6% of adolescents suffer from weekly chronic pain in at least two sites^3^. Girls generally experience more pain than boys do. The prevalence of pain increases with age, and pain in childhood or adolescence is known as a significant predictive factor for pain in the adulthood^4,5^. Although longitudinal studies regarding the risk factors for chronic pain in children and adolescents are rare, factors such as stress, critical life events, lack of leisure hours, high expectations of school officials, separation from parents, frequent change of residence, and bullying are among the causes in this age group^6–10^.

Continuous experience of pain among adolescents may result in serious negative impacts such as widespread disability, sleep problems, missing school, poor school performance, low self-esteem, and withdrawal from social activities^11,12^. In addition, population-based longitudinal studies following youth into adulthood have shown that children with chronic pain are at an increased risk of suffering from mental health disorders later in the adulthood^13^.

In light of the high likelihood that without effective treatment, chronic pain persists into adulthood^5^, and as chronic pain in adolescence may disturb the attainment of developmental outcomes in the adulthood^12^, pain management interventions have gained more and more attention regarding the treatment of chronic pain during adolescence.

Based on the biopsychosocial model, chronic pain is never a mere sensory perception but a complex biopsychosocial condition affected by a wide range of psychosocial factors^14^. Accordingly, lack of attention to the psychosocialfactors can lead to inefficiencies in the treatment of pain. During adolescence, peer and school stressors such as harassment by peers, schoolwork pressure, inappropriate behaviors of teachers as well as principals, and school rules are associated with negative psychological outcomes such as depression, irritability, and nervousness^15,16^. Moreover, during this developmental period, some adolescents may resort to using anger and aggression in order to quest for autonomy, identity, and independence. High levels of stress and anger may, in turn, lead to inability to successfully cope with pain^17,18^. Therefore, it seems that psychological skills training such as stress management, effective communication, and anger management in addition to physical management of pain are important interventions to reduce pain and promote quality of life in adolescents with chronic pain. Despite this, there are some conflicting results. For example, Shearer et al., in their recent review, found no clear evidence for the effectiveness of relaxation training and other psychological interventions on the management of neck pain^19^. Hechler et al., in a study on children and adolescents with high average pain intensity (greater 6 on the NRS), showed that children and adolescents who were treated as outpatients compared to inpatients had less clinically significant changes in their pain intensity and disability^20^. However, there are major limitations such as geographical distance and long waiting lists, preventing most adolescents from receiving face-to-face chronic pain management services^21^. Palermo et al., in their meta-analytic review, advocatedthe necessity of identifying the most relevant and effective components of psychological interventions and considering if they can be adapted for delivery via innovative methods such as mHealth. Also, they highlighted the lack of multi-dimensional assessment of functioning in adolescents with chronic pain^21^.

On the other hand, although some research has shown the positive effects of chronic pain interventions, these effects have not been qualitatively explained. Integration of research findings from quantitative and qualitative approaches in the same study may provide better understanding of pain management than either approach alone. While quantitative methods may demonstrate the statistical significance/power of the pain management program, qualitative methods can provide contextual information that colors the experiences of adolescents^22^. More broadly, pain management occurs in social environments with specific cultural contexts and personal value systems that affect how individuals manage their pain^23^. Qualitative interviews help researchers to produce a more complete picture of pain management strategies adopted by adolescents, to explain the findings of the quantitative study in more depth, and to explain how adolescents think about this phenomenon^22^. The present mixed-method study was conducted to evaluate a smartphone-based pain management application regarding the feasibility, adherence, participant satisfaction, and effectiveness on pain intensity and quality of life in adolescents with chronic pain. The findings in the quantitative stage of the study were explained by the qualitative interviews conducted with a sub-group of adolescents.

## Methods

### Quantitative stage

#### Sampling and recruitment

The sample included 128 adolescents who met the ICD-11 criteria of chronic pain^24^. These adolescents were selected from the baseline sample of our previous study, in which a multistage clustering sampling approach was used to identify adolescents with chronic pain in Shiraz^11^. Recruitment of adolescents was performed by sending advertisement to all adolescents with chronic pain in the previous study. Adolescents who responded to the advertisement were assessed for eligibility before being included into the present study. The following inclusion criteria were applied: age 12–19 years old, willingness to participate in the study, diagnosis of chronic pain according to the ICD-11 Criteria^24^ assessed by pain specialists, and access to smartphone devices and mobile Internet connectivity. Adolescents were excluded if they had a pain history of < 3 months, a history of chronic medical illnesses not related to pain (e.g. asthma), and a history of mental disorders or developmental disorders based on parents’ reports, as well as experiencing a very stressful event recently or during the study period and being reluctant to continue contributing to the study.

#### Study design

A randomized controlled trial study was conducted on 128adolescents in Shiraz, Iran. Before the start of the program, adolescents were provided with the full written information about the trial and signed an informed consent form. After they signed the consent form, they were instructed to complete the questionnaires asking about demographic variables, the level of their pain over the previous 2 weeks, and their actual level of quality of life (T0).Then, adolescents were allocated to either intervention or control conditions. In order to minimize the risk of data contamination and bias, we chose to use a cluster randomization with schools as the units of randomization (rather than individual adolescents) so that two schools in each delivery area were assigned to the intervention and two schools to the control condition. An independent observer not involved in this study, using a coin toss, performed random assignment of schools. No education was given to the adolescents in the control group. However, adolescents allocated to the intervention group received access to the mobile app version of the pain management program, available on Android operating systems. The smartphone-based pain management program was a self-guided intervention including four weekly core modules: (1) pain education, (2) effective communication, (3) stress management, and (4) anger management. The duration of 4 weeks for the intervention in the present study was selected according to the study of Connelly et al. (2006)^25^. Researchers have also reported typical length of psychological interventions for children and adolescents with chronic pain of 3 to 4 weeks^26,27^.

Each module consisted of 3–5 lessons that provided education and teach pain management and/or psychological skills. Pain education focused on the gate control theory of pain as well as the link between chronic pain and psychological factors such as dysfunctional cognitions (e.g., catastrophizing), negative emotions (e.g., fear of pain, stress, and anger), and the process of chronification. Moreover, it focused on the activity-rest cycling, staying active, graded exposure, and the gradually increase of involvement in daily activities. The module of pain education explained the link between activity avoidance and pain; it also motivated adolescents to maintain their own level of physical activity despite pain and to set goals for pleasant activities that they have stopped doing due to their pain. Effective communication module explained the importance of effective communication and strategies that can promote effective verbal and non-verbal communication among adolescents. Stress management module explained the bidirectional relationship between pain and stress as well as providing adolescents with various stress management techniques and exercises (e.g., progressive muscle relaxation, imagination exercises, and diaphragmatic breathing). Anger management module helped adolescents to better understand the relationship between pain and anger and use anger management exercises. Each intervention module took about 30–45 min to complete. Once adolescents finished the modules, they were asked to take brief quizzes and complete the assignments corresponding with skills taught in the modules (e.g., staying active, daily relaxation techniques, etc.). Adolescents were encouraged to practice the skills in each assignment and provide feedbacks on the assignments through text messages in WhatsApp (voluntary). Adolescents could gain access to the next module only by answering the brief quizzes at the end of each module. Upon completion of each module, adolescents were allowed access to the next module. Adolescents were contacted weekly by a psychiatric nurse through text messages and/or phone calls. The psychiatric nurse assessed adolescents’ understanding about the modules and assignments and answered their questions about the content of the modules. Immediately at the end of the fourth week (T1) and three months later (T2) the instruments for pain and quality of life were reapplied and the post-treatment and follow-up scores were obtained. Evaluators were not informed of participants’ treatment assignment.

In addition to the demographic assessment (age, sex, and parents’ occupational and educational level),the following outcome measures were assessed:

1. Pain intensity was assessed with the Numeric Rating Scale (NRS:0 (no pain) to 10 (worst imaginable pain)). Adequate psychometric properties have been reported^28^.
2. Adolescents’ quality of life was measured using the PedsQL 4.0 Generic Core Scale. It is a 23-item self-report questionnaire from 0 (never) to 4 (almost always), designed to measure physical, emotional, social, and school functioning of the child during the previous 4 weeks. In order to obtain the total score, each answer was rescaled to 0–100 scale (0 = 100, 1 = 75, 2 = 50, 3 = 25, 4 = 0) and the total score was comprised of the average of all items in the questionnaire. Higher scores represented better quality of life. Reliability (Cronbach's α = 0.87) as well as discriminant, criterion, and content validity of the Persian version of the instrument were confirmed^29,30^.

The feasibility of the program was assessed using the percentage of eligible adolescents who were enrolled and retained in the study. We defined the study feasible if 70% of the adolescents were adherent to the study^31^.

The number of modules and the assignments that adolescents completed were used as the definition of adherence to the pain management program. Full adherence was defined as completing all the modules and providing feedbacks on all the assignments. In order to measure the level of satisfaction with the pain management program, the client satisfaction questionnaire adapted to Internet-based interventions (CSQ-I) was used^32^. It consisted of 8 items answered on a four-point Likert scale ranging from 1 (does not apply to me) to 4 (does totally apply to me). Hence, the total score of the scale varied from 8 to 32. The Persian version of CSQ-I demonstrated an excellent internal consistency in the present study (Cronbach’s alpha = 0.93). In the present sample, the construct validity of the Persian version of the CSQ-I was confirmed by significant correlations of the CSQ-I score and changes in the scores of emotional functioning(r = 0.39, *p* < 0.01), social functioning (r = 0.26, *p* < 0.03), and school functioning (r = 0.53, *p* < 0.000) between T1 and T2.

### Sample size

Based on the results of a previous study^33^, the mean difference = 1.21, standard deviations (S1 = 2.1, S2 = 1.8), and application of MedCalc software, the calculated sample size was 55 per group to provide 90% power in a two-tailed test with alpha set at 0.05. With an estimated attrition rate of 25%, we planned to include 137 adolescents total.

### Statistical analysis

Descriptive statistics, such as means, standard deviations (SDs), and frequencies as well as percentages were used to assess the demographic characteristics of the sample, and the feasibility, adherence, and satisfaction with the pain management program. Student’st-test for independent samples and χ^2^ tests were performed to compare the groups concerning demographic variables. To test whether smartphone-based pain management program leads to significant improvements in pain intensity and quality of life, repeated measures ANOVA was performed. Effect size was calculated by η2 and Cohen’s d. As suggested by Cohen, effect sizes are categorized as small (d = 0.2), medium (d = 0.5), and large (d = 0.8)^34^. Pearson’s correlation coefficients were calculated between the CSQ-I score and changes in the scores of different dimensions of quality of life between T1 and T2.

### Qualitative stage

This stage of the study used conventional content analysis with purposive sampling. The participants were selected with maximum diversity from both sexes and were assigned to the intervention and control groups. The interviews were conducted in the students’ school. After providing verbal explanations and obtaining informed consent from the adolescents and their parents for participation in the study and recording their voices, the interviews were held in line with the ethical considerations of research. Each interview lasted between 20 and 45 min. Data were collected using semi-structured interviews. Some interview questions included: “How do you cope when in pain?”;“What do you like to do when in pain?”;“What would you like others to do for you when you are in pain?”;“What measures do you take to control your pain?”;and “How do you cope when your pain persists for a long time?”.

The qualitative data were analyzed as they were being collected using qualitative content analysis in OneNote-2013. The conventional qualitative content analysis approach proposed by Graneheim and Lundman was used^35^. Data were immediately recorded, transcribed verbatim, and reviewed several times. Carrying out an analysis required the researcher to submerge in the data. This process began by listening to the participants’ voice and continued with the review of the data several times. For the analysis, first, the text was fully read once, and then it was read line by line for a second time, and the key statements or words were identified. Next, the codes with similar meanings were put in one cluster, and the clusters with relevant meanings formed one category. Each category was given a title that contained all the titles in the group. Then, this group and the categories were assigned to larger categories to the extent possible. The purpose of creating larger categories was to achieve new knowledge, improve the perceptions, and offer a full description of the phenomena. The data rigor was assessed by Guba and Lincoln’s criteria^36^.

After this stage, the quantitative and qualitative data were compared with each other and combined; the results of these two stages were assessed in terms of homogeneity or heterogeneity.

### Ethical approval

The study was conducted in accordance with the human subjects’ protection principles (Declaration of Helsinki) and was approved by the Ethics Committee of the Shiraz University of Medical Sciences (IR.SUMS.REC.1398.1099). All the adolescents and their parents were informed about the research project and an optional withdrawal from the study. The data were collected anonymously. Written informed consent form was signed by all the adolescents and their parents. As soon as the data collection had come to an end, the educational program of pain management was provided to the adolescents in the control group.

## Results

### Quantitative stage

Of the total adolescents who met the ICD-11 criteria of chronic pain in our previous study^6^, 137adolescentsmet the eligibility criteria in the present study. Of the 137 eligible adolescents (n_intervention_ = 68, n_control_ = 69) who started the study, 128 (93.43%) completed it. Of 68 adolescents starting the smartphone-based pain management program (intervention group), 64 (94.11%) completed the program: 2 adolescents were excluded because they did not return the follow-up questionnaires, and 2 adolescents were excluded because they did not want to continue the study. Of these 64 adolescents, 50 adolescents (50/64, 78.12%) fully adhered to the program by completing all the modules and providing feedbacks on all the assignments. Those who did not fully adhere to the program (n = 14) completed all the modules but did not provide feedback on some or all the assignments.

In the control group, 69 adolescents started the study and 64 (92.75%) completed it. Five adolescents had to be excluded from the study because they did not return the post-test or follow-up questionnaires.

The mean age of the adolescents was 13.73 years old [standard deviation (SD) = 1.71], and about 54% of adolescents were female. There was no significant differences between two groups regarding mean age (T (1, 126) = 0.51; *p* = 0.60). Also, no significant differences were found regarding sex (X^2^ = 1.54, df = 1, *p* = 0.21), mother’s education (X^2^ = 0.78, df = 1, *p* = 0.37), father’s education (X^2^ = 0.16, df = 1, *p* = 0.42), mother’s job (X^2^ = 1.82, df = 2, *p* = 0.40), or father’s job (X^2^ = 2.49, df = 2, *p* = 0.28) (Table 1).

**Table 1:** Sample description and analysis of group differences (chi-square, *t*-tests).

	Application group (n = 64)	Control group (n = 64)	T (df)/X^2^ (df)
**Variables**			
Age (Mean ± SD)	13.81 ± 1.72	13.65 ± 1.72	T (1/126) = 0.51^ ns^
**Sex, n (%)**			
Female	38 (55.1%)	31 (44.9%)	X^2^ (1) = 1.54 ns
Male	26 (44.1%)	33 (55.9%)	
**Mother's education, n (%)**			
High school certificate	33 (54.1%)	28 (45.9%)	X^2^ (1) = 0.78 ns
University degree	31 (46.3%)	36 (53.7%)	
**Father's education, n (%)**			
High school certificate	18 (52.9%)	16 (47.1%)	X^2^ (1) = 0.16 ns
University degree	46 (48.9%)	48 (51.1%)	
**Mother's job, n (%)**			
Worker/employer	22 (56.4%)	17 (43.6%)	X^2^ (2) = 1.82 ns
Retired	0	1 (100%)	
Unemployed	42 (47.7%)	46 (52.3%)	
**Father's job, n (%)**			
Worker/employer	61 (52.1%)	56 (47.9%)	X^2^ (2) = 2.49 ns
Retired	1 (25%)	3 (75%)	
Unemployed	2 (28.6%)	5 (71.4%)	

Means and SDs with Cohen’s d effect sizes are presented in Table 2. The results of one-way repeated measures ANOVAs showed that there were significant main effects of time on ratings of pain intensity (F[2/252] = 7.41, *p* < 0.001), emotional functioning (F[2/252] = 5.30, *p* < 0.006), social functioning (F[2/252] = 9.43, *p* < 0.001), and school functioning (F[2/252] = 19.01, *p* < 0.001). However, physical functioning (F[2/252] = 2.22, *p* < 0.11) did not show a significant time effect. There was also a significant main effect of group on ratings of pain intensity (F[1/126] = 5.49, *p* < 0.02), emotional functioning (F[1/126] = 6.76, *p* < 0.01), social functioning (F[1/126] = 8.60, *p* < 0.004), and school functioning (F[1/126] = 4.63, *p* < 0.03) but not on ratings of physical functioning (F[1/126] = 0.53, *p* < 0.46) (Table 3). This means that ignoring the effect of time, there were significant differences among the groups regarding marginal means of pain intensity, emotional functioning, social functioning, and school functioning.

**Table 2:** Pain intensity and Quality of Life Dimensions, Means (SDs) and Effect sizes (Cohen’s d).

Measures	Groups	Pretreatment Mean (SDs)	Posttreatment Mean (SDs)	Follow-up Mean (SDs)	Pre-post effect size, d	Pre-follow up Effect size, d
Pain intensity	Application	4.04(2.49)	2.90(2.32)	3.10(2.21)	0.47	0.39
	Control	4.26(2.86)	4.20(2.53)	4.45(2.64)	0.02	− 0.06
**Quality of life**						
Physical functioning	Application	44.89(9.05)	47.52(8.06)	47.96(10.26)	0.30	0.32
	Control	45.32(9.96)	46.53(9.04)	46.61(6.34)	0.12	0.15
Emotional functioning	Application	54.10(12.88)	61.56(8.63)	60.17(10.41)	0.68	0.51
	Control	55.73(9.89)	56.25(7.71)	55.01(11.76)	0.05	0.06
Social functioning	Application	52.96(15.55)	62.34(5.03)	59.81(7.27)	0.81	0.56
	Control	54.76(12.13)	56.32(10.77)	53.04(11.04)	0.13	− 0.14
School functioning	Application	48.82(11.11)	59.96(12.13)	54.21(10.16)	0.95	0.50
	Control	50.62(9.98)	53.35(9.38)	50.62(9.61)	0.28	0.00

**Table 3:** One-way repeated measures ANOVAs, F-ratios, *p* values, and partial ɳ^2^.

Measures	F(df)	P <	ɳ2
**Between subjects**			
Group			
Pain intensity	5.49(1/126)	0.02	0.04
Quality of life			
Physical functioning	0.53(1/126)	0.46	0.004
Emotional functioning	6.76(1/126)	0.01	0.05
Social functioning	8.60(1/126)	0.004	0.06
School functioning	4.63(1/126)	0.03	0.03
**Within subjects**			
Time			
Pain intensity	7.41(2/252)	0.001	0.05
Quality of life			
Physical functioning	2.22(2/252)	0.11	0.01
Emotional functioning	5.30(2/252)	0.006	0.04
Social functioning	9.43(2/252)	0.001	0.07
School functioning	19.01(2/252)	0.001	0.13
Time*group			
Pain intensity	8.12(2/252)	0.001	0.06
Quality of life			
Physical functioning	0.34(2/252)	0.70	0.003
Emotional functioning	5.04(2/252)	0.007	0.03
Social functioning	7.08(2/252)	0.001	0.05
School functioning	7.04(2/252)	0.001	0.05

The interaction of group × time for pain intensity (F[2/252] = 8.12, *p* < 0.001), emotional functioning (F[2/252] = 5.04, *p* < 0.007), social functioning (F[2/252] = 7.08, *p* < 0.001), and school functioning (F[2/252] = 7.04, *p* < 0.001) was statistically significant (Table 3). This means that the change in these variables over time is different depending on group membership. There was not a significant interaction of group × time for physical functioning (F[2/252] = 0.34, *p* < 0.70) (Table 3).

The satisfaction with pain management application ranged from mean 3.20 (SD = 1.01) on item 6 “The training helped me deal with my problems more effectively” to mean 3.51 (SD = 0.85) on item 8 “I would come back to such a training if I were to seek help again”. The average total CSQ-I score was 26.45 (SD = 6.45) with 21 adolescents (21/64, 32.8%), reporting the highest possible total score. The CSQ-I score was significantly correlated to the changes in the scores of emotional functioning (r = 0.39, *p* < 0.01), social functioning (r = 0.26, *p* < 0.03), and school functioning (r = 0.53, *p* < 0.000) but not physical functioning (*p* > 0.05) between T1 and T2. This indicated that on average, adolescents with more enhancement of emotional, social, and school functioning appeared to be more satisfied with the pain management program.

### Qualitative stage

The participants in this stage included 9 girls and 5 boys, whose demographic details are presented in Table 4.

**Table 4:** The demographic details of the participants in the qualitative stage.

Participant no	Age (year)	Gender	Group
P1	13	Female	Intervention
P2	13	Female	Intervention
P3	14	Female	Intervention
P4	12	Female	Intervention
P5	13	Female	Intervention
P6	13	Female	Intervention
P7	14	Female	Intervention
P8	15	Female	Intervention
P9	17	Male	Control
P10	16	Male	Control
P11	17	Male	Control
P12	12	Male	Control
P13	17	Male	Control
P14	17	Female	Control

According to the interviews, adolescents’ chronic pain management strategies can be classified in three main categories: physical management, psychological management, and interpersonal resources. Table 5 presents the categories and subcategories obtained from the interviews.

- **Physical management**

**Table 5:** The categories, subcategories, and sub-subcategories in the qualitative stage.

Category	Subcategory	Sub-subcategory
Physical management	Medical methods	
	Traditional medicine methods	
Psychological Management	Avoidance-based management	✓ Mental avoidance ✓ Behavioral avoidance
	Courageous management	✓ Resilience ✓ Optimism ✓ Spiritual adaptation
Interpersonal resources	Family support	✓ Sympathy ✓ Presence ✓ Understanding ✓ Helping
	Social support	✓ Peer support ✓ School authorities’ support

The participated adolescents mostly resorted to physical methods of chronic pain management at first, including medical techniques such as analgesics, topical methods such as heat applicaton on the site, and topical medications such as ointments. Other medical techniques used included traditional and herbal medicine, such as applying pepper on the site of pain or drinking herbal brews. They believed that the purpose of these methods was to increase their pain tolerance, shorten the duration of pain, or reduce the severity of pain.

“*First, we use the medications we have at home. Or like, we drink Flixweed seeds with water or herbal brews and thyme tea for stomachache, or for muscular pain, we put pepper on the site, or for muscular pain, we put heat on the site and cover it tightly*” (P14).

Almost all the participants (the control and intervention groups) stated that if these common home remedies failed to work, the next step was to visit a doctor, which might suggest this age group’s trust in medical teams.

*“If the medication given to us by the doctor or our family hasn’t worked, then we go to the doctor”* (P12).

- **Psychological Management**

Psychological methods were participants’ second method of chronic pain management that can be divided into two methods: Avoidance-based management and courageous management.

- Avoidance-based management: In this method, the participants tried to increase their pain tolerance using mental and behavioral techniques or used these methods to distract their mind from the pain. This kind of management, thus, included mental avoidance and behavioral avoidance.
- Mental avoidance: This technique was used by the adolescents in the face of chronic pain. They, thus, avoided thinking about their pain or tried to think about other things. They called these methods distraction techniques.

“I have concerns, but try not to think about them” (P3).

“I thought less about that disease” (P11).

“I occupy myself with other things, and keep myself busy. When you think about the bigger pains you or others have had, the pain becomes more tolerable” (P13).

- Behavioral avoidance: In addition to the mental distraction, the participants also used behavioral techniques to distract themselves. They stated that they wouldfeel more pain if they were constantly thinking about it, evoked negative thoughts, or isolated themselves from others. Therefore, they turned to behaviors that helped them think less about their pain. These behaviors mainly included listening to music, reading, watching TV, socializing withfriends and family, talking with others, and connecting to the nature. Some of their statements revealed these behaviors.

“I kept myself busy. For example, I would reach out to my friends, because being alone makes me depressed, and I feel worse … I do stuff I like. For example, taking care of plants and flowers, doing art craft, connecting to birds, going for a walk in the park, which gives me energy, or feeding animals” (P7).

“In these times, I like to have some fun; like, take a trip, see friends or go to the movies” (P11).

- Courageous management: In this pain management technique, the participants tried to have a greater acceptance of pain and adaptation using positive coping strategies. This kind of management consisted of resilience, optimism, and spiritual adaptation.

Resilience: In the face of chronic pain, some participants revealed that they tried to come to terms with their pain and be patient. In an attempt to endure this pain, this group of adolescents boosted their spirits and told themselves that it was nothing.

“*I have horrifying thoughts about my disease, but I go after solutions ….”* (P10).

“*I remain patient. I mean, I deal with it*” (P9).

- Optimism: Other adolescents treated their pain optimistically by strengthening their positive thoughts and believed that the pain was not permanent, and it would end one day.

“*I must not lose hope. I should not be indifferent, and I should try to make the pain better*” (P12).

“*When we are in pain, we must think about good things, or the fact that we can carry on living*” (P7).

*“I keep thinking that this is not important and will get better*” (P3).

- Spiritual adaptation: Given the cultural and religious context of Iran, some participants were inclined to resort to religious and spiritual matters to cope with their chronic pain. They considered these methods to include Quranic tales or tales surrounding the Imams.

“*I like storytelling. I like my aunt to recite me the Quran or tell me stories about the Imams or Imam Hussein’s story*” (P6).

- **Interpersonal resources**: In addition to the individual resources, the participants also used interpersonal resources to manage their chronic pain. The interpersonal resources included family and social support.
- Family support: According to the participants, family support had a remarkable role in the management of chronic pain. The mother’s role was more significant than other family members, especially for the girls although the father and other family members also played an important role.

“*Family support is very important; that they don’t dish out blame and try to find a solution …”* (P10).

“*If the family provides facilities that make me more comfortable. If they don’t force me to do anything. My sister and brother have an important role in giving me hope, if they play with me”* (P12).

“*My parents, especially my mother, boost my spirits when they sit at my bedside and give me hope, tell me that I’ll be alright and that I can go out with my friends and visit my relatives again, and caress me …”* (P8).

“*To understand and not blame me by saying that I have made too big a deal of it”* (P5).

- Social support: School officials and friends were another group whose support seemed essential in managing chronic pain. As expected, peer group is very important in adolescents’ life. As such, one of the most important support groups consisted of classmates and peers.

“*We expect them to be careful with us, and not joke around and say that you are incapable of doing this or that. To empathize with me and tell me that this is a bridge to my future success*” (P10).

*“My friends to create a happy environment for me, and help me walk, sit down and do my studies. For example, buy me things from the school cafeteria, help me go up the stairs, and bear with me when I’m in a lot of pain*” (P11).

“*I expect my friends to visit me, so I know I’m worth something to them. Talk with me and help me in my studies. Since talking helps me forget the pain*” (P8).

“*I like my friends to play games with me and not worry about my illness or talk behind my back and say how little pain-resistant I am. I don’t like them to come to me and say that I’m exaggerating my pain*” (P5).

The school officials were also another support group. Considering that adolescents spend many hours at school, one of their problems was dealing with their school responsibilities. Therefore, the participants stated that they expected the school officials to support them when they had chronic pain and related diseases and not to be too strict with them about their arrival and leaving hours, doing their assignments, and taking the exams. Some participants stated:

“*If necessary, I expect the school to inform the health teacher and call for an ambulance if it is more severe*” (P6).

“*I expect my teacher not to give me too many assignments and let me rest more*” (P9).

“*The school officials should understand us and empathize too. Like, when we’re late to school, they should understand”* (P10).

## Discussion

The present study was designed and conducted to investigate the effect of a pain management education program on the level of pain and quality of life in adolescents and showed that the designed mobile pain management apppositively affected the adolescents’ pain and quality of life. The quantitative stage results showed that pain management education can be effective in reducing the severity of pain and improving the emotional, social, and school functioning of adolescents but not their physical functioning.

The quantitative stage results in the physical functioning dimension showed no difference between the intervention and control groups. Meanwhile, the results of the qualitative interviews, elaborating on the experience of resorting to physical activities to manage pain in the intervention group, were not consistent with the quantitative results. This finding from the qualitative stage may be attributed to the pain management education, which emphasized the importance of avoiding long-term inactivity in the face of pain. The lack of a significant change in the physical functioning of the intervention group in the quantitative stage can be attributed to the fact that a change has occurred only in the attitude due to the short duration of the education, and no significant change has occurred in terms of behavior with regard to physical functioning. Another noteworthy point was that daily group exercise is one of the important components of the pain management sessions attended; therefore, since daily group exercise was not possible within the framework of mobile-based education, the effect of the provided education on the adolescents’ physical functioning was not significant. Nonetheless, the importance of physical activity was explained for the adolescents in the first pain management educational module. The education provided might prove to be more effective in the physical functioning if the adolescents are more encouraged to carry out daily physical activities (as is the case in the daily programs of health clinics alongside psychotherapy).

Another finding of this study concerns the improvement in the emotional functioning of the intervention group, and the qualitative stage results were somehow in agreement with this finding too. It seems that teaching the importance and management of ineffective thoughts and feelings as well as stress and anger management have been able to effectively improve the emotional functioning of the intervention group. For example, of all the pain management methods at their disposal, the adolescents in the intervention group discussed various methods of coping with negative thoughts and feelings and spiritual adaptation, which was not observed in the control group. Psychological pain management, thus, appears to have been able to abate negative emotions such as fear and stress in the intervention group.

In the social function dimension in the quantitative stage, the intervention group showed significant improvements compared to the control one, which was also confirmed in the qualitative stage. Following the education provided to them in the quantitative stage, the intervention group also had positive changes in terms of school functioning; however, the adolescents’ experience in the qualitative stage did not confirm this finding.

Considering the results of the qualitative and quantitative stages, pain management application appears to have been somewhat effective in improving the severity of pain and different dimensions of quality of life (Table 6). In the pain management education application, the Gate Control Theory and the psychological dimensions of pain were explained to the adolescents in a simple language. Moreover, given that stress, anger, and negative emotions negatively affect pain^37,38^, pain management application provided appears to have been able to somewhat control negative emotions and subsequently helped with pain management in the adolescents. Researchers believe that pain management should be holistic and based on the biopsychosocial model^39^, which means that it does not suffice to address the physical dimension of pain; rather, the psychological and social dimensions should also be considered^38^. In a study conducted by Waite-Jones et al. (2018), the adolescents with arthritis suggested that mobile apps should contain mindfulness exercises and muscle relaxation techniques^40^, and the app in the present study included these trainings in its stress management module.

**Table 6:** Combining the qualitative and quantitative stage results.

Intervention	Quantitative stage results	Qualitative stage results	Interpretation
Investigating the effect of mobile phone-based education on the pain and quality of life of adolescents with chronic pain	No improvement in the physical functioning aspect of quality of life	Challenges in doing home and school assignments (in both the control and intervention groups) No change in exercise activities (in the intervention group) No change in activities such as walking, jogging, going to the bathroom and taking a shower (in both the control and intervention groups)	Agree to some extent
	Improvement in the emotional functioning aspect of quality of life	Feeling fear and horror (in the control group) Feeling sadness and sorrow (in the control and intervention groups) Feeling anger and rage (in the control and intervention groups) No sleep disorder in either of the two groups	Agree to some extent
	Improvement in the social functioning aspect of quality of life	Communication as a pain management technique (in the intervention group) Friends had not stopped being friends (in the intervention group) No experience of being ridiculed by friends (in the control group) Limitations in doing school activities, such as the assignments, going up the stairs, etc. (in both groups) The challenging nature of accompanying friends in daily school activities (in the control group)	Agree to some extent
	Improvement in the school functioning aspect of quality of life	Paying attention to lessons; no instance of forgetting the lessons learnt (in both groups) Problems in doing school work, or absence due to not feeling well (in both groups)	Disagree

There is not much information available about the experiences of adolescents with chronic disorders. In a study investigating the experiences of adolescents with inflammatory bowel disease (IBD), one such an experience was “feeling of being different from peers”^41^. In the present study, one of the concerns of the adolescents was for their friends to view them differently. Furthermore, social support was proposed as one of the interpersonal resources of chronic pain management, which is in line with the results obtained by other researchers^40,41^.

Another study divided the self-management needs of adolescents with sickle cell anemia into four themes. Some subthemes were concerned with the effect of relationship with peers, the importance of self-management, communication establishment, and social support^42^. In a study conducted by Slater et al. (2016) on 16–24-year-old adolescents with permanent musculoskeletal pain, the importance of social support, especially peer groups’ support, was emphasized^43^. These researchers claimed that this kind of support can improve self-esteem in adolescents^44^.

Among the management methods used by the adolescents to cope with chronic diseases, adolescents with IBD believed that they had adopt various methods to cope with their condition that had established over time as they grew up^41^. In the present study, adolescents used methods such as spiritual adaptation which is embedded in the cultural and religious context of Iran^45^. They also used courageous, resilient, or avoidance-based management that although adolescents may not be able to call these methods by their name, the findings show that they are capable of achieving better problem management through appropriate training and counseling. Moreover, the present study was not conducted over a very long time span, and these coping mechanisms may be better revealed if a longer follow-up is considered.

Modern technologies are inherently capable of joining telehealth programs in the not-too-distant future. Such technologies, including apps, can have many advantages for health teams and patients. As such, they can be used as a patient empowering factor^46^. MHealthhas reduced the stigma in children and adolescents and given them a sense of independence and self-efficacy^47–49^; these methods enable the use of interdisciplinary capacities^37^. Despite all the benefits of modern technologies, very few studies have been conducted on apps in the management of chronic diseases, such as chronic pain, especially in adolescents. Recent studies show that many of the adult chronic pain-related apps have no usability or have not been scientifically assessed with regard to the content and suggestions they give to patients^50–52^. Therefore, their efficacy, scientific content and validity should be confirmed in studies^50,52^. Moreover, the assessment, exploration, and confirmation of data from these studies through qualitative assessments provide researchers and those involved with richer information.

By combining qualitative and quantitative data, the present study provides richer and deeper information about the management of chronic pain in adolescents. Since researchers believe that the content of health apps must be assessed by experts of each field, another strength of the present study was app design and its evaluation by a research team member experienced in pain management.

Another important point is that there are very few multidisciplinary pain clinics, and the few existing ones have not been specifically designed for children and adolescents. Moreover, since children and adolescents attend school during the day, they cannot be referred to these clinics for the noted treatments, which further minimizes the possibility of using these methods for children and adolescents. It is, therefore, essential to consider these multidisciplinary activities in designing pain management apps.

According to the results, the satisfaction scores were on average very high, showing that most adolescents reported to be satisfied with the pain management application. Adolescents who had more changes in the scores of emotional, social, and school functioning were more satisfied with the application. However, it has to be noted that most of the CSQ-I items cover the user’s satisfaction with the general quality of the intervention rather than focusing on specific characteristics of the intervention such as usability and simplicity of the intervention content. Future studies should evaluate additional quality dimensions of pain management application that may also be relevant for clinical success.

The majority of the adolescents adhered to the pain management application, indicating its applicability. Perceived ease of use and perceived usefulness of an application can determine the attitude and behavioral intention towards its use, influencing adherence^53^. Adolescents participating in other studies have suggested that apps should be in the form of games so as to be more fun. Also, they should be quiz-styled to strengthen learning activities^40^. The authors of this research also have similar suggestions for designing future chronic pain management apps.

## Conclusion

The present findings confirm the positive effect of education through apps on adolescents’ chronic pain management. Adolescents’ access to smartphones enables them to use these apps for their pain management. The inclusion of concepts such as effective communication skills as well as stress and anger management and considering the psychological dimension of pain management are crucial for chronic pain management education. It is essential for specialists and health team members to address both the psychological and physical dimensions in both virtual and face-to-face programs, such as attending pain clinics, since these dimensions are only partially emphasized in most pain clinics.
